# Intimate partner violence during pregnancy and its association with pregnancy and childbirth complications: A prospective cohort study

**DOI:** 10.1371/journal.pgph.0004311

**Published:** 2025-04-11

**Authors:** Shamsudeen Mohammed, Paul Lokubal, Josephine Akua Ackah Baafi

**Affiliations:** 1 Department of Non-communicable Disease Epidemiology, Faculty of Epidemiology and Population Health, London School of Hygiene and Tropical Medicine, London, United Kingdom; 2 Department of Infectious Diseases and International Health, Faculty of Epidemiology and Population Health, London School of Hygiene and Tropical Medicine, United Kingdom; 3 Department of Population Health, Faculty of Epidemiology and Population Health, London School of Hygiene and Tropical Medicine, London, United Kingdom; Tata Institute of Social Sciences, INDIA

## Abstract

Intimate partner violence (IPV) during pregnancy increases the risk of adverse outcomes for both the woman and foetus. However, there is limited research on its scope and impact in many sub-Saharan African countries. In this study, we investigated the effects of IPV during pregnancy on pregnancy and childbirth complications in Ethiopia. Prospective, longitudinal data from the Performance Monitoring for Action Ethiopia (PMA Ethiopia) Cohort 1 study covering a sample of 2635 women followed up until one year postpartum was used for analysis. Information on IPV during pregnancy and obstetric complications were collected from women at six-week follow-up visits. Multivariable log-binomial regression estimated the risk of antepartum, intrapartum, and postpartum complications associated with IPV of any type, physical IPV and sexual IPV during pregnancy. The prevalence of any IPV type was 13%, only physical IPV was 4.6%, and only sexual IPV was 7.1%. Physical IPV was associated with an increased risk of worsening vision at night during pregnancy (adjusted relative risk [aRR]=2.47, 95% Confidence Interval [95%CI]=1.46 - 4.77), intrapartum haemorrhage (aRR=1.65, 95%CI=1.11 - 2.46), and intrapartum convulsion (aRR=1.98, 95%CI=1.34 - 2.94). Sexual IPV was associated with increased risk for antepartum convulsion (aRR=1.93, 95%CI=1.07 - 3.48), leaked/ruptured membrane (aRR=2.86, 95%CI=1.59 - 5.14), malpresentation (aRR=2.37, 95%CI=1.17 - 4.80), intrapartum convulsions (aRR=1.86, 95%CI=1.16 - 2.98), postpartum haemorrhage (aRR=1.68, 95%CI=1.18 - 2.40) and fever with foul discharge (aRR=2.03, 95%CI=1.40 - 2.93). Overall, the experience of any IPV type increased the risk for the above in addition to migraine, postpartum convulsion, and abnormal vaginal discharge. There is a need to embed IPV sensitisation campaigns in maternal health policies and interventions and to empower women to report cases for timely intervention.

## Introduction

Globally, about 800 preventable maternal deaths occurred daily in 2020 due to pregnancy and childbirth-related causes [1]. Most of these deaths (73%) resulted from direct obstetric complications such as haemorrhage, hypertensive disorders of pregnancy, and sepsis [2]. Intimate partner violence (IPV) is increasingly recognised as a contributing factor to these complications [3,4], increasing the risk of adverse maternal outcomes like haemorrhage, infections, convulsions, prolonged labour, and preterm birth through mechanisms such as physical trauma, chronic stress, and restricted access to maternal healthcare [5–10].

Women in sub-Saharan Africa bear a substantial proportion of the global maternal mortality burden, with over half of maternal deaths in the region attributed to direct obstetric complications [1,2]. For example, a meta-analysis of 70 studies from 19 sub-Saharan African countries revealed that women with hypertensive disorders of pregnancy were 17 times more likely to die from maternal causes compared to those without hypertension [11]. These women also faced increased risks of caesarean section, preterm delivery, and perinatal mortality [11]. This is particularly concerning as around one in ten pregnant women in sub-Saharan Africa experience hypertensive disorders in pregnancy [11,12].

Headache, vaginal bleeding, obstructed labour, retained placenta, and postpartum haemorrhage, are other common pregnancy and childbirth complications in the region. Facility-based studies from Ghana, Nigeria, Kenya, and Ethiopia have highlighted the impact of these complications, with many pregnant women reporting at least one complication [13–17]. In Ethiopia, complications during pregnancy and childbirth are a major contributor to the country’s high maternal mortality rate. Although the maternal mortality rate declined from 401 to 267 deaths per 100,000 live births between 2017 and 2020 [18,19], the rate is still higher than the Sustainable Development Goal target of fewer than 70 deaths per 100,000 live births by 2030 [1].

IPV may further contribute to this burden in Ethiopia, where more than 40% of women [20] and 32% of pregnant women [21] report experiencing violence from either a current or former partner [21–23]. This exposure to violence increases the risk of pregnancy-related complications, yet research on the association between IPV and adverse pregnancy outcomes in Ethiopia is limited and often constrained by methodological challenges. Existing studies [21,24,25] are often limited by small sample sizes, limited geographic coverage, or an emphasis on specific birth outcomes, such as preterm birth and low birth weight, while overlooking the broader impact of IPV during the antepartum and intrapartum periods. For example, one study conducted in Southern Ethiopia found that pregnant women who experienced IPV faced a higher risk of adverse maternal outcomes compared to those who did not [25]. However, the study’s use of a cumulative measure for maternal complications assumed an equal effect of IPV across various obstetric complications, potentially masking the nuanced impact of IPV on specific complications during and after childbirth.

We propose an alternative perspective, hypothesising that the magnitude and impact of IPV on obstetric complications vary depending on the type of complication and the phase of pregnancy at which it manifests - antepartum, intrapartum, and postpartum. The type of IPV also matters. As demonstrated by previous studies in India [26] and Bangladesh [27], the associative effect of IPV on pregnancy complications was higher for physical violence than sexual violence. We, therefore, hypothesise that the effect of intimate partner violence on obstetric outcomes (antepartum, intrapartum, and postpartum) would vary between those experiencing only physical IPV and those experiencing only sexual IPV.

By examining these differences, our study offers a new perspective currently lacking in the existing body of research, thereby strengthening efforts to incorporate IPV prevention mechanisms into maternal health policies and programs. Overall, we aim to investigate the effects of IPV during pregnancy on antepartum, intrapartum, and postpartum obstetric complications among women in Ethiopia, using data from a population-based prospective, longitudinal cohort study.

## Methods

### Study setting

Ethiopia, located in the Horn of Africa, is a low-income country bordered by Sudan, Somalia, Djibouti, Eritrea, and Kenya [28,29]. It is the 10th largest and second most populous African country, with a 2023 population of about 126 million, 85% of whom live in rural areas. Administratively, Ethiopia is divided into 9 National Regional States and two administrative councils (Addis Ababa and Dire Dawa). Addis Ababa is the Capital and largest city. The country is linguistically diverse, with over 80 languages and about 200 dialects [28,29]. Maternal health is a public health concern in Ethiopia, marked by limited access to comprehensive emergency obstetric care services [30]. Pregnancy and childbirth outcomes, such as obstructed labour, haemorrhage, preeclampsia, placenta retention, or puerperal sepsis, remain a concern and often contribute to Ethiopia’s high maternal and infant mortality rates [31–34]. In 2020, the maternal mortality ratio was 267 per 100,000 live births, accounting for 3.6% of global maternal deaths [19,35]. Direct obstetric complications were the primary cause of these deaths [33,34].

### Data source

We used prospective, longitudinal data from the Performance Monitoring for Action Ethiopia (PMA Ethiopia) Cohort 1 study. The design and methods of the cohort have been described in detail elsewhere [23,24]. PMA Ethiopia was conducted in six regions of Ethiopia: Tigray, Oromia, Amhara, SNNPR (Southern Nations, Nationalities and Peoples’ Region), Afar, and Addis Ababa [36,37]. Participants were sampled using a multistage stratified cluster sampling method, with urban-rural and major regions as strata. Using the Central Statistical Agency’s master sample frame, 206 enumeration areas were selected from the regions using probability proportional to size [38]. In October 2019, field personnel conducted a census of all households within the enumeration areas to identify eligible women. Women between the ages of 15 and 49 who were pregnant or had given birth within the previous six months were invited to participate in the panel survey. A total of 32791 women from 32614 households were screened, and 2880 eligible women were identified. Of the 2880 eligible women, 2869 (99.6%) gave informed consent and were enrolled in the survey. Baseline interviews were conducted for the eligible women between September and December 2019 [36]. The pregnant women were followed up at six weeks, six months, and one year postpartum. For women postpartum at baseline, the baseline survey and six-week postpartum interview were completed at enrolment. The present analysis was restricted to data collected at baseline and six-week follow-up. Women with multiple births (n=52, 1.7%) were excluded because they presented a unique risk to the outcomes investigated. The final sample comprised 2635 women.

### Intimate partner violence

A modified version of the Conflicts and Tactics Scale (CTS) was used to measure physical and sexual violence during pregnancy [39]. Physical violence was assessed with seven questions asked of postpartum women during the six-week follow-up interview. These questions aimed to determine if, at any time during the pregnancy, their husband or partner had engaged in the following behaviours: (1) pushing, shaking, or throwing something at them, (2) slapping them, (3) twisting their arm or pulling their hair, (4) punching them with a fist or an object that could cause harm, (5) kicking, dragging, or physically assaulting them, (6) attempting to choke or intentionally burn them, and (7) threatening or attacking them with a knife, gun, or any other weapon. Sexual violence was assessed using three questions that asked the postpartum women whether, at any time during the pregnancy, their husband or partner had (1) physically forced them to engage in sexual intercourse against their will, (2) physically forced them to perform other unwanted sexual acts, or (3) used threats or pressure to coerce them into having sex when they did not want to, without resorting to physical force. The response options for all questions were *“Yes”* or *“No”.* Women who reported experiencing at least one act of physical or sexual violence or both were considered to have experienced *“IPV of any type”*. However, if a woman reported only one or more acts of physical violence, she was classified as a victim of physical intimate partner violence (physical IPV). Similarly, if a woman reported only one or more acts of sexual violence, she was classified as a victim of sexual intimate partner violence (sexual IPV).

### Outcome

The pregnancy and childbirth complications we investigated were classified into three groups: antepartum complications (disorders or complications during pregnancy); intrapartum complications (complications during labour and childbirth); and early postpartum complications (complications occurring <48 hours after giving birth). Using a questionnaire, field staff asked women at the six-week follow-up interviews following childbirth if they experienced any health problems during the pregnancy, including severe headache with blurred vision (Migraine), oedema of the face/feet/body, convulsion or fits, vaginal bleeding before birth, abnormal vaginal discharge (foul smelling/dark), or worsening vision at night. For intrapartum complications, women were asked whether they experienced any of the following problems during the childbirth: severe bleeding, leaking or ruptured membrane and no labour pain for >24 hours, malpresentation or malposition (the feet/hand came out first or baby lied transversely during pregnancy), prolonged labour (>12 hours), and convulsions or fits. To assess postpartum complications, mothers were asked whether they experienced any of the following problems within the first 24 hours after giving birth: severe/heavy bleeding, retained placenta (more than 30 minutes), high fever with foul/smelly discharge or lower abdominal pain, and convulsion/fits. Response options were “Yes”, “No”, “Do not know”, and “No response”. The few women who responded “Do not know”, and “No response” were excluded. The data were collected by the PMA after obtaining oral informed consent from all study participants and seeking ethical approval from the Institutional Review Boards of Johns Hopkins Bloomberg School of Public Health in Baltimore and Addis Ababa University in Ethiopia. Ethical approval was not required for this analysis as we used anonymised publicly available secondary data.

### Statistical analysis

Summary statistics (frequencies and percentages) were calculated to examine baseline sociodemographic characteristics. The prevalence of IPV of any type, physical IPV, and sexual IPV was estimated, and their associations with participants sociodemographic characteristics were determined using Pearson’s χ2 test. Missing data (<5%) were not imputed, given its minimal expected impact on estimates. The proportion of women who suffered antepartum, intrapartum, and postpartum complications was visualised, and Pearson’s χ2 test of independence was used to assess the bivariate association between the experience of intimate partner violence and antepartum, intrapartum, and postpartum complications. We hypothesised that any effect of intimate partner violence on pregnancy and childbirth outcomes would differ between those who experienced only physical IPV and those who experienced only sexual IPV. Therefore, multivariable log-binomial regression models were fitted using a log link function to determine the relative risk of antepartum, intrapartum, and postpartum complications associated with IPV of any type, as well as physical IPV and sexual IPV during pregnancy. Estimates were adjusted for maternal age, region, marital status, household income, number of previous births, residence (urban-rural), and maternal education. Covariates were carefully selected based on a literature search, our understanding of intimate partner violence and pregnancy and childbirth outcomes, and bivariate evidence from our data. All analyses accounted for the multistage sampling design by applying individual sampling weights to ensure the representativeness of the target population. These weights accounted for differential probabilities of selection and nonresponse across primary sampling units (PSUs). To address clustering effects due to the multistage sampling and ensure accurate variance estimation, strata and PSUs were specified to minimise biases associated with clustering within enumeration areas. These adjustments were performed using Stata’s svyset and svy commands. All analyses were conducted using Stata 17.

## Results

### Participants characteristics

The analytical sample consisted of 2635 women, ages 15 to 48. In all, 11.2% of the participants were adolescents, 16.7% were 35 or older, and 30.2% were between the ages of 25 and 29 (Table 1). Most (95.2%) participants were married, and 75.6% lived in rural areas. Only 4.2% had higher education, and 40.3% never attended school. Of the 2635 participants, 39.1% lived in low-income households, and 31.5% had four or more previous births.

**Table 1:** Baseline characteristics of participants, overall and by type of intimate partner violence.

	Overall sample	Intimate partner violence (IPV)		
		IPV of any type	Physical IPV	Sexual IPV
	2635	341 (13.0)	109 (4.6%)	176 (7.1%)
**Age**				
<20	295 (11.2)	30 (10.2)	9 (3.3)	14 (5.2)
20-24	617 (23.4)	89 (14.5)	35 (6.2)	36 (6.4)
25-29	795 (30.2)	102 (12.9)	36 (5.0)	56 (7.4)
30-34	490 (18.6)	53 (10.8)	18 (3.9)	29 (6.3)
≥35	438 (16.7)	67 (15.2)	12 (3.0)	40 (9.8)
**Marital status**				
Married	2505 (95.2)	314 (12.5)	105 (4.6)	163 (6.9)
Unmarried	127 (4.8)	25 (19.9)	5 (4.5)	11 (9.9)
**Educational level**				
Never attended	1062 (40.3)	138 (13.0)	43 (4.4)	73 (7.3)
Primary	1070 (40.6)	141 (13.2)	46 (4.8)	69 (6.9)
Secondary	393 (14.9)	49 (12.5)	10 (2.9)	32 (8.4)
Higher	111 (4.2)	13 (11.8)	10 (9.1)	2 (1.9)
**Residence**				
Urban	644 (24.4)	68 (10.6)	31 (5.2)	28 (4.7)
Rural	1991 (75.6)	273 (13.7)	78 (4.4)	147 (7.9)
**Household wealth level**				
Low	1030 (39.1)	150 (14.5)	36 (3.9)	87 (9.0)
Middle	515 (19.5)	70 (13.6)	22 (4.7)	32 (6.7)
High	1090 (41.4)	122 (11.2)	52 (5.1)	57 (5.5)
**Number of previous births**				
None	499 (18.9)	70 (14.0)	26 (5.7)	33 (7.1)
1	577 (21.9)	78 (13.5)	26 (4.9)	37 (6.9)
2-3	729 (27.7)	79 (10.8)	26 (3.8)	39 (5.7)
4+	830 (31.5)	115 (13.8)	32 (4.3)	67 (8.5)
**Region**				
Tigray	182 (6.9)	29 (15.8)	8 (4.7)	13
Afar	52 (2.0)	2 (3.2)	1 (2.7)	0 (0.0)
Amhara	536 (20.3)	52 (9.7)	13 (2.6)	32 (6.3)
Oromia	1163 (44.1)	135 (11.6)	70 (6.3)	50 (4.7)
SNNP	600 (22.8)	119 (19.9)	14 (2.9)	79 (14.2)
Addis Ababa	103 (3.9)	5 (4.8)	4 (3.5)	0 (0.0)

Counts and percentages are weighted.

### Prevalence of intimate partner violence

Overall, 13.0% of study participants experienced IPV of any type (sexual, physical, or both) during pregnancy; 7.1% experienced only sexual IPV, and 4.6% experienced only physical IPV (Table 1). Unmarried women had a higher prevalence of IPV during pregnancy than married women, but there was no difference in their experiences of physical (*p* = 0.98) and sexual (*p* = 0.20) IPV. Women from low-income households had a higher prevalence of sexual violence than those from middle- and high-income households. The prevalence of IPV of any type (*p* = 0.03), including sexual (*p* = 0.02) and physical (*p* = 0.04) IPV, varied across the six regions where participants were recruited. However, there was no evidence of a difference in IPV prevalence based on a woman’s educational level, age, place of residence, or parity (*p* > 0.05 for all IPV types) .

### Prevalence of adverse antepartum, intrapartum, and postpartum outcomes

**Fig 1:**
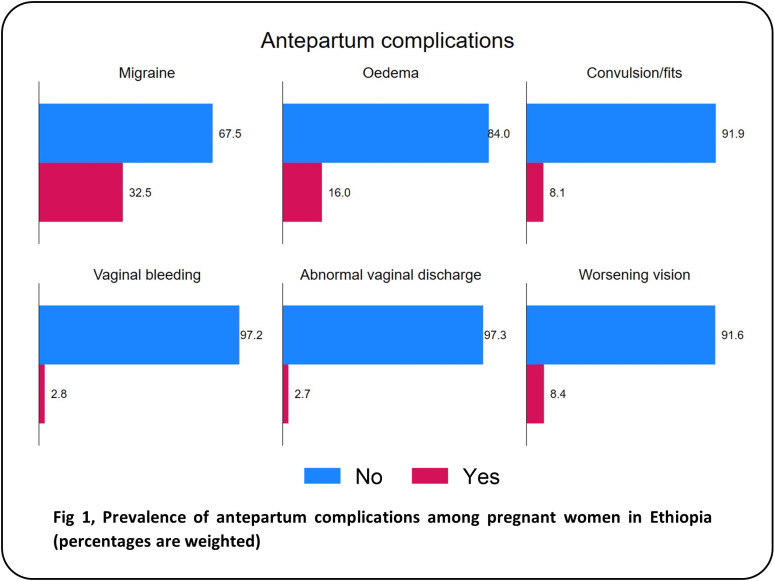
Prevalence of antepartum complications among pregnant women in Ethiopia (percentages are weighted).

**Fig 2:**
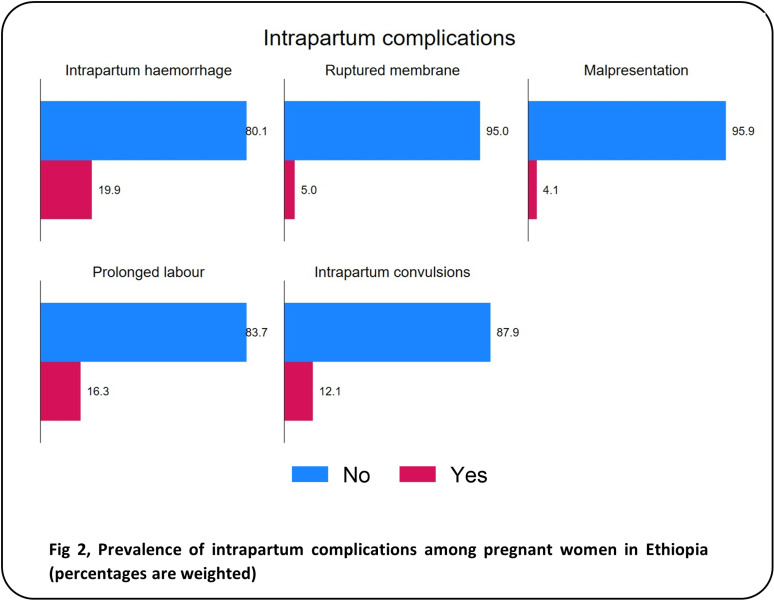
Prevalence of intrapartum complications among pregnant women in Ethiopia (percentages are weighted).

**Fig 3:**
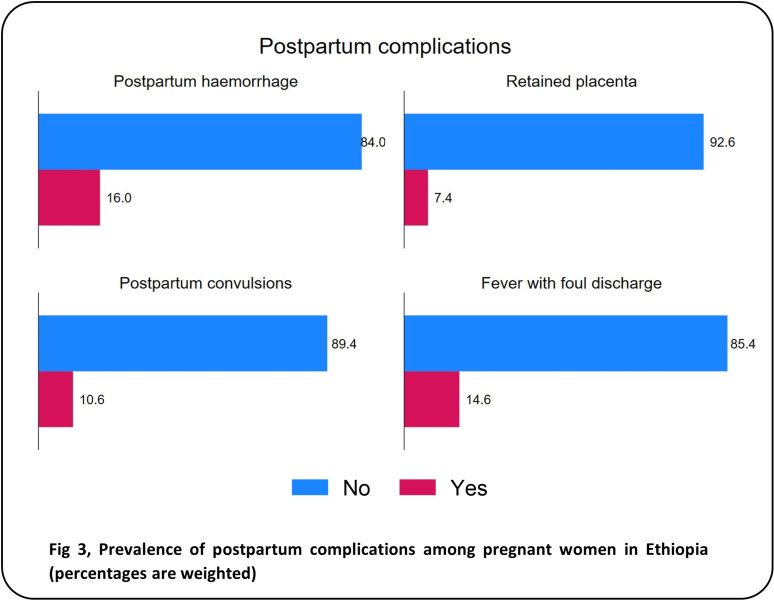
Prevalence of postpartum complications among pregnant women in Ethiopia (percentages are weighted).

During pregnancy (Fig 1), the most frequently reported disorder was migraine headache (32.5%), followed by oedema of the feet or face (16.0%), night-time vision impairment (8.4%), and convulsions or fits (8.1%). Only 2.8% and 2.7% of the women reported experiencing vaginal bleeding and abnormal vaginal discharge during pregnancy, respectively. During childbirth (Fig 2), the most frequent complications reported were intrapartum haemorrhage (19.9%), prolonged labour (16.3%) and intrapartum convulsions (12.1). Malpresentation occurred in 4.1% of the participants. Additionally, 5.0% of the participants experienced a leaking or ruptured membrane and no labour pain for >24 hours. Of the four postpartum complications examined (Fig 3), postpartum haemorrhage (16.0%) and fever with foul discharge (14.6%) after birth were the most frequently reported complications. Postpartum convulsions or fits and placental retention after birth were experienced by 10.6% and 7.4%, respectively. The distribution of the sociodemographic characteristics across the obstetric complications is presented in S1 Table.

## Association between intimate partner violence (IPV) during pregnancy and adverse antepartum, intrapartum, and postpartum obstetric outcomes

Unadjusted results of the association between IPV and adverse antepartum, intrapartum, and postpartum obstetric outcomes is presented in S1 Table.

### Intimate partner violence

**Fig 4:**
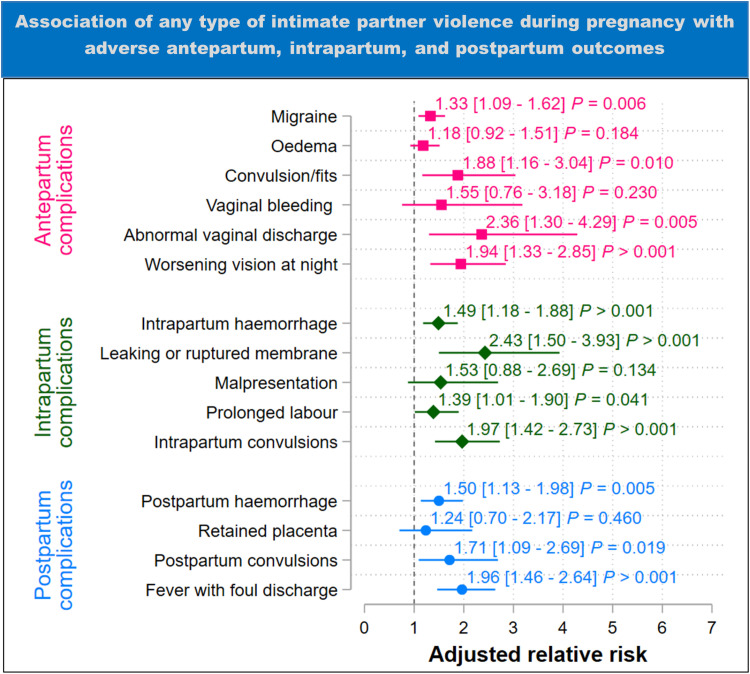
Results of adjusted analysis of the association between any type of intimate partner violence during pregnancy and adverse antepartum, intrapartum, and postpartum outcomes (estimates are adjusted for maternal age, region, marital status, household income, number of previous births, residence (urban-rural), and maternal education). The plot shows the adjusted relative risk with 95% confidence intervals in square brackets. ****P**** = p-value.

Fig 4 shows that after adjusting for potential confounders, the experience of any type of IPV increased the risk of experiencing migraine headaches by 1.33 times (95%CI 1.09 – 1.62), convulsion or fits by 1.88 times (95%CI 1.16 – 3.04), abnormal vaginal discharge by 2.36 times (95%CI 1.30 – 4.29), and vision impairment at night by 1.94 times (95%CI 1.33 – 2.85) than not experiencing any type of IPV.

For intrapartum complications, the experience of any type of IPV was associated with 1.49 times (95%CI 1.18 – 1.88) the risk of intrapartum haemorrhage, 2.43 times (95%CI 1.50 – 3.93) the risk of leaking or ruptured membrane 24 hours before labour, 1.39 times (95%CI 1.01 – 1.90) the risk of prolonged labour, and 1.97 times (95%CI 1.42 – 2.73) the risk of intrapartum convulsions during labour and childbirth than not experiencing any type of IPV.

For postpartum complications, the experience of any type of IPV was associated with 1.50 times (95%CI 1.13 – 1.98) the risk of postpartum haemorrhage, 1.71 times (95%CI 1.09 – 2.69) the risk of postpartum convulsions, and 1.96 times (95%CI 1.46 – 2.64) the risk of fever with foul discharge after birth than not experiencing any type of IPV.

### Physical intimate partner violence

**Fig 5:**
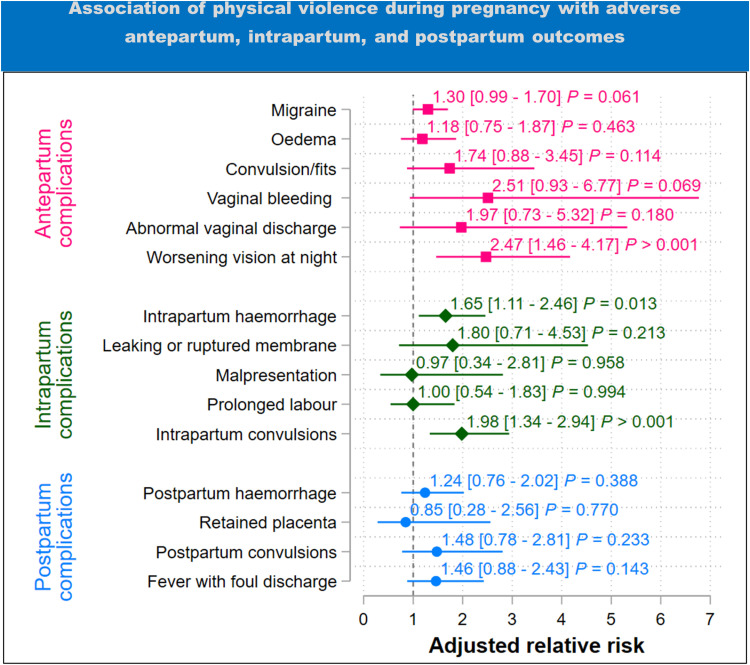
Results of adjusted analysis of the association between sexual violence during pregnancy and adverse antepartum, intrapartum, and postpartum outcomes (estimates are adjusted for maternal age, region, marital status, household income, number of previous births, residence (urban-rural), and maternal education). The plot shows the adjusted relative risk with 95% confidence intervals in square brackets. ****P**** = p-value.

In the subgroup of women who experienced only physical IPV during pregnancy (Fig 5), we found some evidence that the experience of only physical IPV increased the risk of a woman experiencing migraine headaches by 1.30 times (95%CI 0.99 – 1.70), vaginal bleeding by 2.51 times (95%CI 0.93 – 6.77), and night vision impairment by 2.47 times (95%CI 1.46 – 4.17) than not experiencing any IPV.

For intrapartum complications (Fig 5 ), the experience of only physical IPV was associated with 1.65 times (95%CI 1.11 – 2.46) the risk of intrapartum haemorrhage and 1.98 times (95%CI 1.34 – 2.94) the risk of intrapartum convulsions than not experiencing any IPV. There was no evidence of an association between the experience of only physical IPV and postpartum complications.

### Sexual intimate partner violence

**Fig 6:**
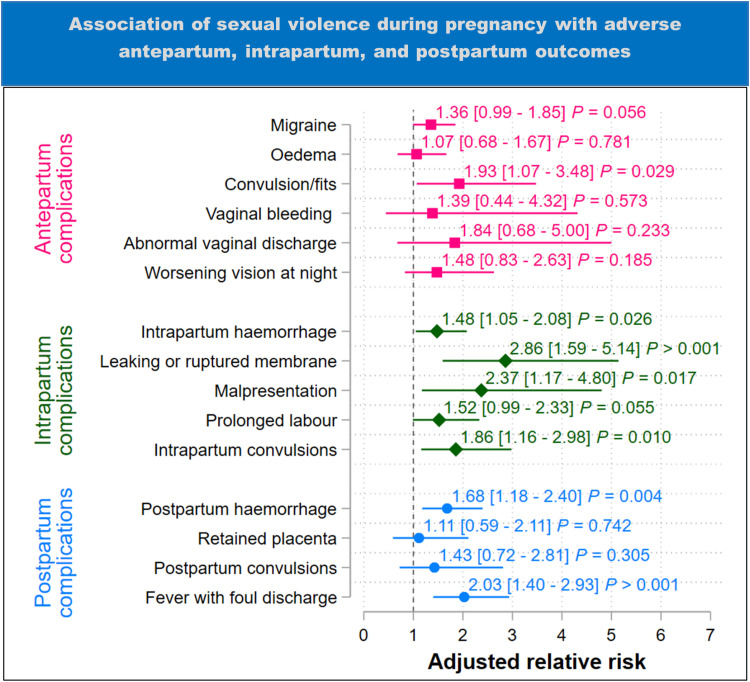
Results of adjusted analysis of the association between sexual violence during pregnancy and adverse antepartum, intrapartum, and postpartum outcomes (estimates are adjusted for maternal age, region, marital status, household income, number of previous births, residence (urban-rural), and maternal education). The plot shows the adjusted relative risk with 95% confidence intervals in square brackets. ****P**** = p-value.

In the subgroup of women who experienced only sexual IPV during pregnancy (Fig 6), we found some evidence that the experience of only sexual IPV increased the risk of a woman experiencing migraine headaches by 1.36 times (95%CI 0.99 – 1.85) and convulsions or fits by 1.93 times (95%CI 1.07 – 3.48) than not experiencing any IPV.

For intrapartum complications (Fig 6), the experience of only sexual IPV was associated with 1.48 times (95%CI 1.05 – 2.08) the risk of intrapartum haemorrhage, 2.86 times (95%CI 1.59 – 5.14) the risk of leaking or ruptured membrane 24 hours before labour, 2.37 times (95%CI 1.17 – 4.80) the risk of malpresentation, 1.52 times (95%CI 0.99 – 2.33) the risk of prolonged labour, and 1.86 times (95%CI 1.16 – 2.98) the risk of convulsions during labour and childbirth than not experiencing any IPV.

For postpartum complications, the experience of only sexual IPV was associated with 1.68 times (95%CI 1.18 – 2.40) the risk of postpartum haemorrhage and 2.03 times (95%CI 1.40 – 2.93) the risk of fever with foul discharge than not experiencing any IPV.

## Discussion

In this longitudinal cohort study, we investigated the effect of intimate partner violence (IPV) during pregnancy on adverse pregnancy and obstetric outcomes in Ethiopia. Our findings showed that 13% of the women experienced any form of IPV (physical or sexual), with 4.6% reporting only physical IPV and 7.1% reporting only sexual IPV. These prevalence estimates are lower than those reported in previous studies from Ethiopia and elsewhere, including a meta-analysis of predominantly cross-sectional studies [21,23,40]. Several factors may explain this variation. Our study’s focus on physical and sexual IPV, to the exclusion of psychological IPV, likely contributed to the lower rates, as psychological IPV is known to be more common among pregnant women[23,41–44]. Differences in study designs may also explain the observed variations. While most previous studies collected IPV information several months after birth, our IPV data were collected mainly within the first six weeks postpartum, minimising recall bias. Additionally, cultural perceptions of IPV may have influenced the observed variations [45]. Studies have reported that IPV is often viewed as a normal part of marriage in Ethiopia, with men using violence to maintain authority [46,47]. Many women accept this practice, which can lead to reluctance to report IPV.

Our findings confirmed the hypotheses of a potential differential effect of IPV across various obstetric complications. Women who experienced any IPV had a higher risk of pregnancy and obstetric complications, but the effects varied depending on the type of complication. We observed that the type of IPV mattered, with physical and sexual IPV showing somewhat distinct associations with different complications. Physical IPV increased the risks for worsening vision at night during the antepartum period as well as intrapartum haemorrhage and convulsions during childbirth but had no effect on postpartum complications. In contrast, sexual IPV was associated with convulsions in the antepartum period, all intrapartum complications, and postpartum haemorrhage and fever with foul discharge in the postpartum period. For intrapartum haemorrhage and convulsion, where both physical and sexual IPV had significant effects, the magnitude of the effect was slightly stronger for physical IPV.

The mechanisms through which IPV during pregnancy contributes to complications in pregnancy and childbirth are complex and diverse. One possible pathway involves the stress response triggered by IPV [48–50]. This type of stress, including what is known as “battered woman syndrome,” [49] is characterised by re-experiencing the trauma of abuse even when it is not occurring, along with symptoms such as hypervigilance, disrupted relationships, and a distorted body image. The physiological response to this IPV-induced stress can elevate cortisol levels, weaken immune function, and disrupt hormonal balance [51–53], all of which may contribute to a higher risk of complications during pregnancy and childbirth [51–53]. The stress response triggered by IPV differs from other forms of stress due to its chronic nature and the intimate context in which it occurs.

It is possible that physical IPV increased the risk of antepartum and intrapartum haemorrhage through abdominopelvic trauma, potentially causing injuries like placental abruption or uterine rupture. Physical IPV can also result in direct head trauma [54], which may explain the higher risk of neurological symptoms, including impaired vision, migraines, and convulsions, among pregnant women in this study who experienced physical IPV. Sexual IPV has been consistently linked to an increased risk of sexually transmitted infections (STIs) and reproductive tract infections [55–57]. In a systematic review of 26 studies, 22 reported an association between IPV and STIs [55]. These infections can impair maternal health and may explain the higher risk of postpartum haemorrhage, convulsions, fever with foul discharge, and migraine among the participants who suffered sexual violence. Sexual IPV in the form of forced or coerced vaginal penetration can result in weakened cervix and compromised foetal membranes. This physical trauma might explain the increased risk of membrane rupture or leakage and haemorrhage among victims of sexual violence.

Women who experience IPV also face additional barriers to accessing timely and adequate prenatal care [58,59]. Fear, lack of control, and the abuser’s control tactics can prevent women from seeking regular prenatal visits or disclosing their experiences of violence to healthcare providers [59]. Inadequate prenatal care can result in missed opportunities for early detection and management of potential complications, increasing the risk of adverse maternal outcomes [58,60]. Some women may turn to substance abuse as a coping mechanism, which can have detrimental effects on their health and foetal development (placental insufficiency, foetal growth restriction) [58–60]. Concurrent mental health issues such as depression, anxiety, and post-traumatic stress disorder (PTSD) can lead to poor self-care, poor nutrition, and noncompliance with medical advice [41,58,60]. Also, IPV often isolates women from their support systems, including family, friends, and healthcare providers [59]. Lack of social support during pregnancy can lead to elevated stress levels, impaired coping mechanisms, and decreased access to resources and information necessary for maintaining good maternal health [58,59].

Our findings are consistent with those of previous studies [54,61–64]. However, the majority of previous studies focused on the impact of IPV on overall adverse pregnancy outcomes. Our study provides a new perspective by investigating how IPV affects specific complications during pregnancy, labour and childbirth, and the immediate postpartum period. Our findings that the effects of IPV differ across these critical stages, provide useful information for targeted interventions and the development of protocols for risk assessments. In settings such as Ethiopia, where IPV may be underreported due to socio-economic and cultural factors [65], routine screening for IPV during pregnancy is critical. Integrating IPV screening into antenatal and maternal health services [64] can facilitate early identification of at-risk women and enable timely interventions. It is also important to train healthcare providers to recognise and respond to IPV sensitively, thereby ensuring that women feel safe and supported when disclosing their experiences. Additionally, providing comprehensive support services, including mental health counselling, [66] can help minimise the long-term health consequences of IPV and improve maternal outcomes.

Our study has several strengths. Firstly, we provide an update on the prevalence of IPV during pregnancy and obstetric complications (antepartum, intrapartum and postpartum). We also provide evidence of the varying risks that IPV poses to the different complications women face before, during and after childbirth. Furthermore, our sample captured more Zones, improving the representativeness and generalisability of our estimates. IPV was assessed using a validated scale [39]. While our findings are based largely on prospective cohort data, where measurement of IPV preceded the outcomes, minimising recall bias and reverse causality, a small group of women (n=277) were enrolled 5-9 months postpartum. The PMA-Ethiopia surveys that provided data for this analysis did not collect data on psychological or emotional IPV, and the characteristics of perpetrators.

## Conclusion

Women’s experience of IPV in pregnancy is associated with obstetric complications in the antepartum, intrapartum and postpartum period. As pregnancy is a critical window in women’s sexual and reproductive life, with effects on maternal morbidity and mortality, policies should aim to protect women from IPV. This could include sensitisation and awareness campaigns that empower women to report cases of IPV for timely management. Health workers should be trained to be able to screen for and manage or refer cases of IPV to prevent maternal complications. We recommend further research to explore the prevalence of other forms of IPV, including psychological IPV and its effect on obstetric complications in Ethiopia.

## Supporting information

S1 Table Bivariate association between intimate partner violence during pregnancy and antepartum, intrapartum, and postpartum complications. (DOCX)
