# Supplementary material for: Intimate partner violence during pregnancy and its association with pregnancy and childbirth complications: A prospective cohort study
Source: PLOS Glob Public Health. 2025 Apr 11;5(4):e0004311. doi: 10.1371/journal.pgph.0004311 (PMC11990748; doi:10.1371/journal.pgph.0004311)
Supplement: S1 Table — (DOCX) [file pgph.0004311.s001.docx]

**S1 Table.** **Bivariate association between intimate partner violence during pregnancy and antepartum, intrapartum, and postpartum complications**

|  |  |  |  | **Antepartum complications** | | |  |  |  |  |  |  |
| --- | --- | --- | --- | --- | --- | --- | --- | --- | --- | --- | --- | --- |
|  | **Migraine** | | **Oedema** | | **Convulsion** | | **Vaginal bleeding** | | **Abnormal vaginal discharge** | | **Vision problems** | |
|  | No | Yes | No | Yes | No | Yes | No | Yes | No | Yes | No | Yes |
|  | n(%) | n(%) | n(%) | n(%) | n(%) | n(%) | n(%) | n(%) | n(%) | n(%) | n(%) | n(%) |
|  |  |  |  |  |  |  |  |  |  |  |  |  |
| **Overall intimate partner violence** | P = 0.01 | | P 0.27 | | P = 0.004 | | P = 0.17 | | P = 0.007 | | P < 0.001 | |
| No | 1513 (68.9) | 683 (31.1) | 1851 (84.3) | 344 (15.7) | 2036 (92.8) | 159 (7.2) | 2138 (97.4) | 56 (2.6) | 2140 (97.6) | 52 (2.4) | 2029 (92.5) | 164 (7.5) |
| Yes | 185 (58.0) | 134 (42.0) | 261 (81.9) | 58 (18.1) | 273 (85.5) | 46 (14.5) | 306 (95.8) | 13 (4.2) | 303 (94.8) | 17 (5.2) | 272 (85.1) | 48 (14.9) |
|  |  |  |  |  |  |  |  |  |  |  |  |  |
| **Physical intimate partner violence** | P = 0.02 | | P = 0.25 | | P = 0.17 | | P = 0.10 | | P = 0.15 | | P = 0.006 | |
| No | 1513 (68.9) | 683 (31.1) | 1851 (84.3) | 344 (15.7) | 2036 (92.8) | 159 (7.2) | 2138 (97.4) | 56 (2.6) | 2140 (97.6) | 52 (2.4) | 2029 (92.5) | 164 (7.5) |
| Yes | 57 (55.2) | 46 (44.8) | 81 (79.5) | 21 (20.5) | 90 (88.3) | 12 (11.7) | 97 (94.2) | 6 (5.8) | 98 (95.1) | 5 (4.9) | 86 (84.3) | 16 (15.7) |
|  |  |  |  |  |  |  |  |  |  |  |  |  |
| **Sexual intimate partner violence** | P = 0.11 | | P = 0.85 | | P = 0.007 | | P = 0.45 | | P = 0.39 | | P = 0.07 | |
| No | 1513 (68.9) | 683 (31.1) | 1851 (84.3) | 344 (15.7) | 2036 (92.8) | 159 (7.2) | 2138 (97.4) | 56 (2.6) | 2140 (97.6) | 52 (2.4) | 2029 (92.5) | 164 (7.5) |
| Yes | 95 (58.7) | 67 (41.3) | 138 (84.9) | 24 (15.1) | 137 (84.5) | 25 (15.5) | 156 (96.1) | 6 (3.9) | 157 (96.3) | 6 (3.7) | 142 (87.6) | 20 (12.4) |
|  |  |  |  |  |  |  |  |  |  |  |  |  |
|  |  |  |  | **Intrapartum complications** | | |  |  |  |  |  |  |
|  | **Intrapartum haemorrhage** | | **Leaking or ruptured membrane** | | **Malpresentation** | | **Prolonged labour** | | **Intrapartum convulsion** | | **Caesarean birth** | |
|  | No | Yes | No | Yes | No | Yes | No | Yes | No | Yes | No | Yes |
|  | n(%) | n(%) | n(%) | n(%) | n(%) | n(%) | n(%) | n(%) | n(%) | n(%) | n(%) | n(%) |
|  |  |  |  |  |  |  |  |  |  |  |  |  |
| **Overall intimate partner violence** | P < 0.001 | | P = 0.001 | | P = 0.19 | | P = 0.04 | | P < 0.001 | | P = 0.39 | |
| No | 1784 (81.4) | 408 (18.6) | 2098 (95.7) | 94 (4.3) | 2097 (96.1) | 85 (3.9) | 1854 (84.4) | 342 (15.6) | 1964 (89.4) | 233 (10.6) | 1123 (90.4) | 120 (9.6) |
| Yes | 225 (71.3) | 91 (28.7) | 286 (89.8) | 32 (10.2) | 300 (94.3) | 18 (5.7) | 252 (78.8) | 68 (21.2) | 247 (77.3) | 73 (22.7) | 106 (87.6) | 15 (12.4) |
|  |  |  |  |  |  |  |  |  |  |  |  |  |
| **Physical intimate partner violence** | P = 0.04 | | P = 0.28 | | P = 0.96 | | P = 0.97 | | P = 0.002 | | P = 0.62 | |
| No | 1784 (81.4) | 408 (18.6) | 2098 (95.7) | 94 (4.3) | 2097 (96.1) | 85 (3.9) | 1854 (84.4) | 342 (15.6) | 1964 (89.4) | 233 (10.6) | 1123 (90.4) | 120 (9.6) |
| Yes | 72 (70.7) | 30 (29.3) | 94 (92.8) | 7 (7.2) | 99 (96.2) | 4 (3.8) | 86 (84.3) | 16 (15.7) | 82 (80.2) | 20 (19.8) | 42 (92.4) | 3 (7.6) |
|  |  |  |  |  |  |  |  |  |  |  |  |  |
| **Sexual intimate partner violence** | P = 0.01 | | P = 0.001 | | P = 0.02 | | P = 0.07 | | P = 0.001 | | P = 0.05 | |
| No | 1784 (81.4) | 408 (18.6) | 2098 (95.7) | 94 (4.3) | 2097 (96.1) | 85 (3.9) | 1854 (84.4) | 342 (15.6) | 1964 (89.4) | 233 (10.6) | 1123 (90.4) | 120 (9.6) |
| Yes | 113 (70.8) | 47 (29.2) | 144 (88.7) | 18 (11.3) | 148 (91.7) | 13 (8.3) | 126 (77.6) | 36 (22.4) | 125 (76.7) | 38 (23.3) | 42 (80.7) | 10 (19.3) |
|  |  |  |  |  |  |  |  |  |  |  |  |  |
|  |  |  |  | **Postpartum complications** | | |  |  |  |  |  |  |
|  | **Postpartum haemorrhage** | | **Retained placenta** | | **Postpartum convulsions** | | **high fever with foul/smelly discharge** | |  |  |  |  |
|  | No | Yes | No | Yes | No | Yes | No | Yes | -- | -- | -- | -- |
|  | n(%) | n(%) | n(%) | n(%) | n(%) | n(%) | n(%) | n(%) | -- | -- | -- | -- |
|  |  |  |  |  |  |  |  |  |  |  |  |  |
| **Overall intimate partner violence** | P = 0.003 | | P = 0.42 | | P = 0.008 | | P < 0.001 | | -- | -- | -- | -- |
| No | 1867 (85.1) | 327 (14.9) | 2025 (92.9) | 155 (7.1) | 1983 (90.4) | 212 (9.6) | 1912 (87.3) | 278 (12.7) | -- | -- | -- | -- |
| Yes | 247 (77.1) | 73 (22.9) | 289 (91.0) | 29 (9.0) | 264 (82.5) | 56 (17.5) | 231 (73.0) | 85 (27.0) | -- | -- | -- | -- |
|  |  |  |  |  |  |  |  |  |  |  |  |  |
| **Physical intimate partner violence** | P = 0.46 | | P = 0.63 | | P = 0.36 | | P = 0.21 | | -- | -- | -- | -- |
| No | 1867 (85.1) | 327 (14.9) | 2025 (92.9) | 155 (7.1) | 1983 (90.4) | 212 (9.6) | 1912 (87.3) | 278 (12.7) | -- | -- | -- | -- |
| Yes | 84 (81.9) | 19 (18.1) | 97 (94.6) | 6 (5.4) | 89 (86.7) | 14 (13.3) | 84 (82.1) | 18 (17.9) | -- | -- | -- | -- |
|  |  |  |  |  |  |  |  |  |  |  |  |  |
| **Sexual intimate partner violence** | P = 0.001 | | P = 0.56 | | P = 0.18 | | P < 0.001 | | -- | -- | -- | -- |
| No | 1867 (85.1) | 327 (14.9) | 2025 (92.9) | 155 (7.1) | 1983 (90.4) | 212 (9.6) | 1912 (87.3) | 278 (12.7) | -- | -- | -- | -- |
| Yes | 120 (73.6) | 43 (26.4) | 146 (91.4) | 14 (8.6) | 138 (84.7) | 25 (15.3) | 113 (71.3) | 46 (28.7) | -- | -- | -- | -- |
